# ‘Fit for surgery’: the relationship between cardiorespiratory fitness and postoperative outcomes

**DOI:** 10.1113/EP090156

**Published:** 2022-06-05

**Authors:** George A. Rose, Richard G. Davies, Ian R. Appadurai, Ian M. Williams, Mohamad Bashir, Ronan M. G. Berg, David C. Poole, Damian M. Bailey

**Affiliations:** ^1^ Neurovascular Research Laboratory Faculty of Life Sciences and Education University of South Wales Pontypridd UK; ^2^ Department of Anaesthetics University Hospital of Wales Cardiff UK; ^3^ Department of Surgery University Hospital of Wales Cardiff UK; ^4^ Department of Biomedical Sciences Faculty of Health and Medical Sciences University of Copenhagen Copenhagen Denmark; ^5^ Department of Clinical Physiology and Nuclear Medicine University Hospital Copenhagen – Rigshospitalet Copenhagen Denmark; ^6^ Centre for Physical Activity Research University Hospital Copenhagen – Rigshospitalet Copenhagen Denmark; ^7^ Departments of Kinesiology Anatomy and Physiology Kansas State University Manhattan KS USA

**Keywords:** cardiorespiratory fitness, mortality, oxygen transport, physical activity, surgery

## Abstract

**New Findings:**

- **What is the topic of this review?**
  The relationships and physiological mechanisms underlying the clinical benefits of cardiorespiratory fitness (CRF) in patients undergoing major intra‐abdominal surgery.
- **What advances does it highlight?**
  Elevated CRF reduces postoperative morbidity/mortality, thus highlighting the importance of CRF as an independent risk factor. The vascular protection afforded by exercise prehabilitation can further improve surgical risk stratification and postoperative outcomes.

**Abstract:**

Surgery accounts for 7.7% of all deaths globally and the number of procedures is increasing annually. A patient's ‘fitness for surgery’ describes the ability to tolerate a physiological insult, fundamental to risk assessment and care planning. We have evolved as obligate aerobes that rely on oxygen (O_2_). Systemic O_2_ consumption can be measured via cardiopulmonary exercise testing (CPET) providing objective metrics of cardiorespiratory fitness (CRF). Impaired CRF is an independent risk factor for mortality and morbidity. The perioperative period is associated with increased O_2_ demand, which if not met leads to O_2_ deficit, the magnitude and duration of which dictates organ failure and ultimately death. CRF is by far the greatest modifiable risk factor, and optimal exercise interventions are currently under investigation in patient prehabilitation programmes. However, current practice demonstrates potential for up to 60% of patients, who undergo preoperative CPET, to have their fitness incorrectly stratified. To optimise this work we must improve the detection of CRF and reduce potential for interpretive error that may misinform risk classification and subsequent patient care, better quantify risk by expressing the power of CRF to predict mortality and morbidity compared to traditional cardiovascular risk factors, and improve patient interventions with the capacity to further enhance vascular adaptation. Thus, a better understanding of CRF, used to determine fitness for surgery, will enable both clinicians and exercise physiologists to further refine patient care and management to improve survival.

## 1 INTRODUCTION

Surgery is amongst the leading risk factors for mortality and has been estimated to account for 7.7% of all deaths globally (Nepogodiev et al., 2019). By 2030, it is estimated that one‐fifth of people aged 75 years and older in the United Kingdom alone will undergo surgery (Fowler et al., 2019). Therefore, to better understand and mitigate this risk, we need to consider not just the disease or surgical procedure, but also the phenotypical response and ability to cope with the physiological insult posed by major surgery. Furthermore, prophylactic intervention targeting modifiable risk factors prior to surgery, a process known as ‘prehabilitation’, requires investigation and optimisation. This review explores our relationship with oxygen (O_2_), the elixir of life, and how its transport and use in the human body determines ‘fitness for surgery’.

## 2 ORIGIN OF O_2_ AND OUR DEPENDENCY ON OXIDATIVE METABOLISM

When the solar system emerged 4.6 billion years ago (Dickerson, 1978), Earth's atmosphere was devoid of O_2_, a vast difference compared with the modern day atmospheric inspired fraction of 20.93%. The emergence of life, likely originating in alkaline thermal vents at the bottom of the oceans, initially gave rise to the domains of archaea and bacteria (Miller & Bada, 1988). Approximately 1.5 billion years ago, cyanobacteria began to release O_2_ into the atmosphere (Nisbet & Sleep, 2001). The organic compounds that emerged from the ‘primordial soup’ were photosynthetic, capturing solar radiation and creating the organic molecule glucose. In turn, the O_2_ released into the atmosphere signalled a major evolutionary event, arguably described by two oxidation ‘pulses’, the Great Oxidation Event and the Neoproterozoic Event, or as a progressive evolution, the Great Oxidation Transition (Lyons et al., 2014). This gave rise to atmospheric O_2_ and the evolution of O_2_‐dependent organisms, from primitive eukaryotic unicellular structures performing metabolism, locomotion and reproduction to present day *Homo sapiens*.

**FIGURE 1:**
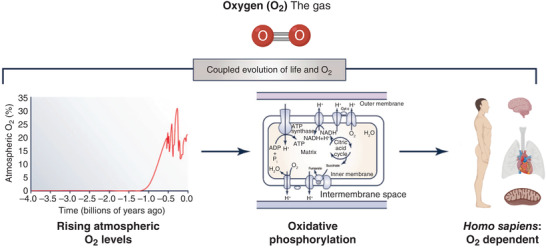
The production of oxygen (O_2_), dependency of mitochondrial oxidative phosphorylation upon O_2_, and the evolution of *Homo sapiens* to support O_2_ delivery. Adapted from Bailey (2019)

Figure 1 describes the production of paleo‐atmospheric O_2_ and the entire dependency of the respiring mammalian cell for the constancy of electron flow, with molecular O_2_ serving as the terminal electron acceptor in mitochondrial oxidative phosphorylation. *Homo sapiens*, like all mammals, has a remarkable ability to harness O_2_, allowing a rapid turnover of adenosine triphosphate (ATP) and affording cells, tissue and organs a coordinated stasis sustaining life. Mammalian evolution has thus produced a structural, functional and physiological organisation that efficiently coordinates the convective delivery and diffusive uptake of O_2_, essential for successful life.

## 3 FROM MOUTH TO MITOCHONDRIA: CONVECTIVE AND DIFFUSIVE DETERMINANTS OF O_2_ TRANSPORT

Early measurements describing O_2_ uptake (${\dot{V}}_{O_{2}}$) in humans at the onset of intense movement were conducted by Hill and Lupton (1923) and demonstrated a rapid and exponential response, as skeletal muscle has the capacity to increase rate of metabolism by an astounding 50‐ to 100‐fold above its resting requirements. This challenges a rapid delivery of O_2_ to the mitochondrial inner membrane for use as the terminal electron acceptor, whereby oxidative phosphorylation generates ATP. O_2_ is transported by convection, which describes its movement within the airways and circulation‐driven aero‐ and hydrostatic pressure gradients, and by diffusion, the passive movement down a concentration gradient such as between the alveolar compartment and pulmonary capillary bed and between the systemic microcirculation and tissue.

**FIGURE 2:**
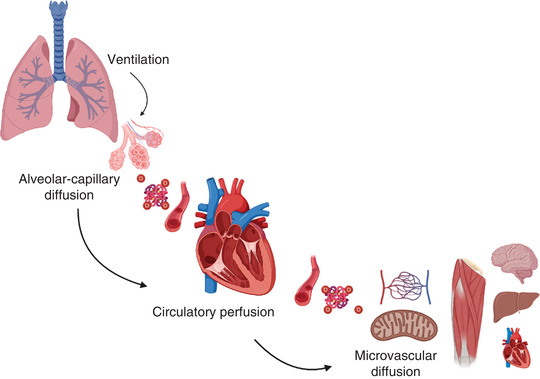
The oxygen (O_2_) transport system characterised by pulmonary ventilation, alveolar–capillary diffusion, circulatory perfusion driven by the cardiovascular system and diffusion across the microcirculatory capillary beds. The volume of O_2_ transport, described by Fick's principle, is determined by the product of convective (cardiac output) and diffusive O_2_ transport terms (and is the product of cardiac output and the difference between the O_2_ content of arterial and venous blood)

Figure 2 illustrates the major organs and processes, both convective and diffusive, that describe the ‘O_2_ cascade’. Following inspiration of air into the lungs, O_2_ diffuses down a concentration gradient at the alveolar–capillary membrane, minimally dissolves in plasma and predominantly binds with haemoglobin (Hb), an allosteric protein with affinity for four molecules of O_2_. Deoxygenated venous blood is therefore saturated with O_2_ in the pulmonary capillaries, the concentration of which is proportional to the concentration of Hb, its *P*_50_ and the partial pressure exerted by O_2_ on the plasma at a given temperature (Henry's law). Oxygenated blood then travels the vascular system driven by the heart. This convective component is referred to as ‘O_2_ delivery’ (${\dot{Q}}_{O_{2}}$), the product of cardiac output ($\dot{Q}$) and arterial O_2_ content ($\dot{Q}$ × $C_{aO_{2}}$), and is complete when O_2_ diffuses across the microcirculatory capillary beds and reaches the mitochondrial matrix where it is used as the terminal electron carrier. ${\dot{V}}_{O_{2}}$ as described by Fick's principle is equal to the product of $\dot{Q}$ and the difference between arterial and mixed venous oxygen content ($C_{aO_{2}}$ – $C_{\bar{v}O_{2}}$). Notably, in health, the principal ‘rate limiting’ steps for maximal O_2_ uptake (${\dot{V}}_{O_{2}\max}$) are attributed to the perfusive (${\dot{Q}}_{O_{2}}$) and diffusive components of the cascade (Wagner, 2000).

## 4 METRICS AND MEANING: ASSESSMENT OF CARDIORESPIRATORY FITNESS

**FIGURE 3:**
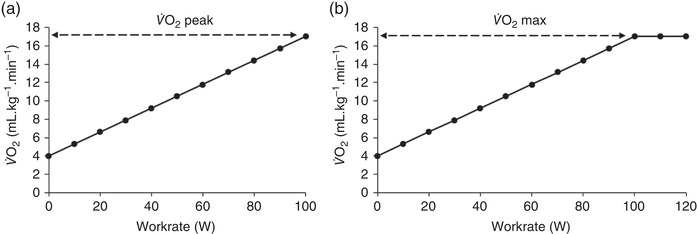
Schematic representation of O_2_ consumption at the limit of exercise tolerance during CPET. (a) ${\dot{V}}_{O_{2}\mathrm{peak}}$ reported as the highest value recorded. (b) ${\dot{V}}_{O_{2}\max}$ idealised as a true highest value with observed plateau present. ${\dot{V}}_{O_{2}\mathrm{peak}}$, peak oxygen consumption; ${\dot{V}}_{O_{2}\max}$, maximal oxygen uptake

**FIGURE 4:**
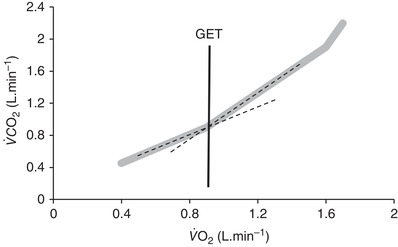
Schematic representation of the *V*‐slope method (Beaver et al., 1986) for estimation of the gas exchange threshold (GET) during CPET. GET is identified at the intersection of two linear sections of the ${\dot{V}}_{CO_{2}}$–${\dot{V}}_{O_{2}}$ relationship, represented by the continuous black line. A further deflection point in the relationship may be observed during the latter stages of CPET and represents respiratory compensation

The advent of breath‐by‐breath measurement technology has allowed us to measure the capacity of the O_2_ transport system and determine metrics describing the magnitude of cardiorespiratory fitness (CRF), which not only describes an individual's ability to perform physical activity, but is linked to cardiovascular health (Ross et al., 2016) and longevity (Blair et al., 1989). Cardiopulmonary exercise testing (CPET) is used to objectively measure the ability of a patient to uptake O_2_ and typically involves an incremental exercise test to symptom‐limited exhaustion. CPET can also identify underlying pathology and evaluate the impact of chronic comorbidities on O_2_ uptake. Recently, the use of CPET has been widely adopted in patients prior to major surgery and approximately 30,000 tests are conducted annually in the UK alone (Reeves et al., 2018). These data are used to support patient care decisions, plan appropriate postoperative critical care, and direct prehabilitation programs aimed at improving CRF (Levett et al., 2018). Three primary metrics describing CRF are typically reported when conducting CPET:

1. Peak oxygen consumption (${\dot{V}}_{O_{2}\mathrm{peak}}$), defined as the ${\dot{V}}_{O_{2}}$ attained during an incremental test to exhaustion, expressed in absolute terms (ml min^−1^) or relative to body mass (ml kg^−1^ min^−1^), which can be subject to allometric scaling, and measured as the highest value recorded, often occurring during the final 20 s of a test. Whilst ${\dot{V}}_{O_{2}\mathrm{peak}}$ is reflective of a patient's ‘best effort’, it may not necessarily reflect a true highest value, defined as ${\dot{V}}_{O_{2}\max}$ with an observed plateau present in the O_2_ uptake work‐rate slope of Hill & Lupton (1923) demonstrated in Figure 3. Controversy exists here, and evidence suggests only a minority of continuous tests, even in young healthy people, yield a measurable plateau (Day et al., 2003; Poole & Jones, 2017). Nevertheless, an exercise test to exhaustion is important since it allows for the site of transport limitation across the O_2_ cascade to be identified (Wagner, 2000).
2. Anaerobic threshold (AT), a submaximal index of CRF defined as the ${\dot{V}}_{O_{2}}$ above which anaerobic metabolism supplements oxidative phosphorylation with additional carbon dioxide (CO_2_) production, creating a deflection point on a plot of pulmonary CO_2_ output *versus* O_2_ uptake (Figure 4). The AT is also commonly reported as a percentage of ${\dot{V}}_{O_{2}\mathrm{peak}}$ or ${\dot{V}}_{O_{2}\max}$. Whilst the AT signifies a transition where increased glycolysis raises the muscles lactate efflux into the blood above its removal rate with associated metabolic acidosis, a multitude of definitions and controversies exist (Poole et al., 2021). Thus in the context of preoperative CPET, AT refers to the gas exchange threshold (GET, sometimes also referred to as the ventilatory threshold), typically measured using the ‘gold standard’ V‐slope (Beaver et al., 1986) method of determination. GET is expressed in ml kg^−1^ min^−1^ or ml min^−1^.
3. The ventilatory equivalent for carbon dioxide ($V_{\mathrm{eqC}O_{2}}$), defined as a ratio of minute ventilation to CO_2_ production and usually reported at the GET. $V_{\mathrm{eqC}O_{2}}$ reflects the composite efficiency of the ventilatory response, including the breathing pattern and adaptive changes in pulmonary gas exchange in response to exercise. Elevated values for $V_{\mathrm{eqC}O_{2}}$ occur in heart failure, respiratory disease and pulmonary hypertension (ATS/ACCP, 2003; Snowden et al., 2010; Sun et al., 2001) consequent to diminished perfusion, ventilation–perfusion mismatching, or diffusion limitation and changes in breathing pattern, which increase dead space ventilation.

## 5 CRF AND SURGERY: LINK TO SURVIVAL

Mortality following major surgery is a significant risk despite progress being made in surgical technologies, anaesthesia and peri‐operative care. In colorectal surgery, mortality is reported at 3.2% within 90 days (NBOCA, 2017) with complication rates above 30% (Lucas & Pawlik, 2014). Similarly, in‐hospital mortality for elective abdominal aortic aneurysm (AAA) repair is 2.9% for open repair and 0.4% for endovascular repair (VSQI, 2017). Furthermore, the insult of major colorectal surgery has been shown to reduce CRF by ∼40%, with hospital stays of 7–9 days, and only 50% of patients regaining preoperative CRF levels after 6 months (Jensen et al., 2011).

Accurate prediction of surgical risk is required to facilitate shared decision making, improve patient outcomes and plan perioperative care. Traditionally, subjective clinical acumen alone was used; however, objective scoring systems are available including the Portsmouth Physiological and Operative Severity Score for the Enumeration of Mortality and Morbidity (P‐POSSUM; Whiteley et al., 1996), American Society of Anaesthesiologists (ASA) physical status, Charlson Comorbidity Index, and measures of cardiac function (Moyes et al., 2013). These systems are generally weak, and complementary ‘biomarkers’ are needed. CRF, a modifiable risk factor, has long been (inversely) associated with all‐cause mortality (Kokkinos et al., 2010; Mandsager et al., 2018), and evidence also suggests that impaired CRF (see Older et al., 1993, below) is associated with reduced survival and increased morbidity following major surgery (Moran et al., 2016; Smith et al., 2013).

The seminal work of Older et al. (1993) first described an association between preoperative CRF and postoperative outcome. They studied 184 elderly patients undergoing elective major intra‐abdominal surgery and established that patients classified as ‘unfit’ exhibited markedly higher mortality rates than those deemed ‘fit’ (18% *vs*. 0.8%, *P* < 0.001). Patients were considered unfit by preoperative CPET if O_2_ uptake at GET was <11 ml O_2_ kg^−1^ min^−1^, a value originally described by Weber and Janicki (1985) that characterised the GET in patients with moderate to severe heart failure. Studies have since used the GET as a measure of CRF, and further supported the inverse association between CRF and postoperative mortality and morbidity in patients undergoing a variety of intra‐abdominal surgeries (Table 1).

**TABLE 1:** Studies demonstrating an association between CRF and postoperative outcome following non‐cardiac intra‐abdominal surgery, adapted from (Moran et al., 2016)

Author	Patients (*n*)	${\dot{V}}_{O_{2}\mathrm{peak}}$ risk threshold (ml kg^−1^ min^−1^)	GET risk threshold (ml O_2_ kg^−1^ min^−1^)	$V_{\mathrm{eqC}O_{2}}$ risk threshold	Risk thresholds defined/adopted	Postoperative outcome
Intra‐abdominal surgery						
Older et al. (1993)	187	Not measured	Yes <11.0	Not measured	Adopted	Hospital mortality
Older et al. (1999)	548	Not measured	Yes <11.0	No	Adopted	Hospital mortality
Wilson et al. (2010)	847	Not measured	Yes <10.9	Yes >34	Adopted	Mortality 90 days
Snowden et al. (2010)	116	No	Yes <10.1	No	Defined	Morbidity: comp
Vascular AAA surgery						
Carlisle and Swart (2007)	130	Yes	Yes	Yes >42	Defined	Mortality: 2 years
Hartley et al. (2012)	415	Yes <15.0	Yes <10.2	Yes >42	Adopted	Mortality: 30 days, 90 days
Prentis et al. (2012)	185	No	Yes <10.0	No	Defined	Morbidity: LoS
Goodyear et al. (2013)	188	Not measured	Yes <11.0	Not measured	Adopted	Mortality: 30 days Morbidity: LoS
Grant et al. (2015)	506	Yes <15.0	Yes <10.2	Yes >42	Adopted	Mortality: 3 years
Rose et al. (2018a)	124	Yes <13.1	No	Yes ≥34	Defined	Mortality: 2 years
Colorectal surgery						
Lai et al. (2013)	269	Not measured	Yes <11.0	Not measured	Adopted	Mortality: 2 years Morbidity: LoS
West et al. (2014b)	136	Yes <16.7	Yes <10.1	Yes >32	Defined	Morbidity: comp
West et al. (2014a)	105	Yes <18.6	Yes <10.6	No	Defined	Morbidity: comp
Wilson et al. (2019)	1375	Not measured	No	Yes >39	Defined	Mortality: 90 days
Upper gastrointestinal surgery						
McCullough et al. (2006)	109	Yes <15.8	No	No	Defined	Morbidity: comp
Nagamatsu et al. (2001)	91	Yes <800 ml	Yes	Not measured	Defined	Morbidity: comp
Moyes et al. (2013)	108	No	Yes <9.0	No	Defined	Morbidity: comp
Patel et al. (2019)	120	Yes <17.0	No	No	Defined	Morbidity: comp

Risk thresholds relate to a level of CRF below which an inferior postoperative outcome has been observed and are either defined from the respective study data or have been adopted from other studies and applied to the study data. Abbreviations: AAA, (open) abdominal aortic aneurysm; comp, complications; GET, gas exchange threshold; LoS, hospital length of stay; $V_{\mathrm{eqC}O_{2}}$_,_ ventilatory equivalent for carbon dioxide; ${\dot{V}}_{O_{2}\mathrm{peak}}$_,_ peak oxygen consumption.

**FIGURE 5:**
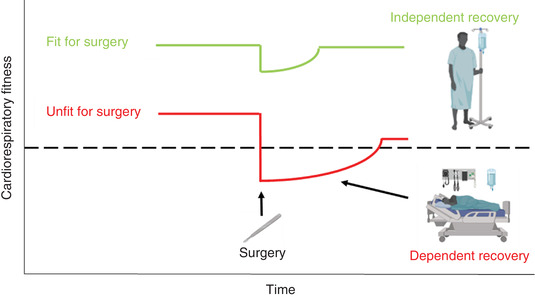
Physiological insult of surgery and potential for change in patient recovery, adapted from Clegg et al. (2013). The green plot represents a patient considered (CRF) ‘fit’ for surgery whereas the red plot represents a patient classified as ‘unfit’. The dashed line represents the cut‐off between independent patient recovery typically requiring ward‐based care, and dependent recovery requiring high dependency unit or intensive care unit admission

A theoretical model (Figure 5) originally developed by Clegg et al. (2013) helps visualise why elevated CRF is associated with improved postoperative outcome. The model describes potential differences in surgical outcome between a hypothetical patient who is unfit for surgery (for example with a GET < 11 ml O_2_ kg^−1^ min^−1^) compared to a patient deemed fit (GET ≥ 11 ml O_2_ kg^−1^ min^−1^). The unfit patient is more likely to require care in a high dependency unit or intensive care unit with a greater likelihood of complications and risk of mortality, whereas the fit patient may experience a normal and faster recovery on the ward.

Given the importance of assessing CRF in clinical practice, the American Heart Association has published a scientific statement promoting CRF as a clinical vital sign (Ross et al., 2016).

## 6 MECHANISTIC LINK BETWEEN CRF AND POSTOPERATIVE OUTCOME

The model presented (Figure 5) presumes the existence of an obligatory baseline level of CRF (such as the threshold values for ${\dot{V}}_{O_{2}\mathrm{peak}}$, GET or $V_{\mathrm{eqC}O_{2}}$ found in Table 1) to survive an increased demand for O_2_ during the perioperative period. If the patient is unable to meet this presumed O_2_ demand, chronic hypoxaemia and limited ${\dot{Q}}_{O_{2}}$ may be responsible for increased morbidity and mortality for any severity of disease. Whilst a detailed mechanistic understanding explaining why impaired CRF is associated with poor postoperative outcome remains to be elucidated, the presence of an O_2_ deficit during the perioperative period is fundamental to this model.

**FIGURE 6:**
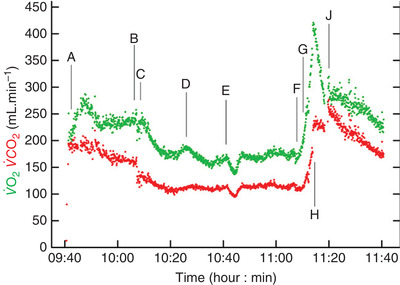
Pulmonary O_2_ uptake during the intraoperative period of a patient undergoing abdominal aortic aneurysm repair, taken from Ciaffoni et al. (2016). Events are represented by knife to skin (A); reduction in ventilator driving pressure (B); aortic clamp applied (C); fall in blood pressure (D); metaraminol (fast‐acting α‐agonist) bolus, infusion rate increased from 2 to 5 ml h^−1^ (E); sequential removal of iliac artery clamps (F, G); increase in ventilator driving pressure (H); and removal of superior retractor restricting rib cage movement (I)

The surgical stress response is characterised by an increased O_2_ demand as demonstrated by Ciaffoni et al. (2016), measured directly (via in‐airway sensors) beginning in the intraoperative period (Figure 6). The underlying mechanisms responsible for the perioperative elevation in ${\dot{V}}_{O_{2}}$ can be explained by complex changes in metabolic demand. These comprise hormonal, haematological and immunological changes, manifest by increased $\dot{Q}$ and O_2_ consumption as the delivery of nutrient and O_2_‐rich blood supports energy processes, tissue repair and protein synthesis (Gillis & Wischmeyer, 2019). Shoemaker et al. (1988) also describe a substantial increase in O_2_ demand, from an average of 110 ml min^−1^ m^−2^ at rest to 170 ml min^−1^ m^−2^ following major surgery, consequent to the strong systemic inflammatory response. Thus, a patient with greater ${\dot{V}}_{O_{2}}$ reserve may help mitigate this cardiovascular burden.

Surgery, is also known to result in oxidative stress with consequent increases in free radical formation (Arsalani‐Zadeh et al., 2011; Bailey et al., 2007, 2006). This is particularly prominent during abdominal surgery given the potential for ischemia–reperfusion, leukocyte activation, mitochondrial dysfunction and concurrent depletion of antioxidants in the postoperative period due to increased consumption (Bailey et al., 2006; Musil et al., 2005; Thomas & Balasubramanian, 2004). During laparoscopic surgery, for example, increases in intra‐abdominal pressure during pneumoperitoneum may cause splanchnic ischemia–reperfusion and subsequent oxidative stress (Leduc & Mitchell, 2006). Furthermore, a reduction in $\dot{Q}$ contributing to decreased O_2_ delivery is observed as systemic venous return is reduced to the right side of the heart and pulmonary venous return reduced to the left side of the heart. The conformational changes in ventricular architecture combine to decrease $\dot{Q}$ and are compounded by an increase in systemic vascular resistance. Ciaffoni et al. (2016) also demonstrated concurrent elevation of CO_2_ production during the intraoperative period, which may be equally important in terms of ‘clearance’ for the maintenance of normal acid–base balance (Bailey et al., 2017) and requires further mechanistic investigation.

The contribution of anaesthesia to the production of reactive oxygen species (ROS) in the perioperative period is also important. O_2_ is one of the most used drugs in anaesthetic practice. Reducing cellular hypoxia is a clinical priority, and thus the sickest patients who are likeliest to suffer the adverse consequences of hyperoxia and ROS formation are the likeliest to receive supplemental O_2_. There is a good case for accepting lower levels of arterial oxygenation to minimise ROS damage in the perioperative period. However, because of genetic variability in susceptibility to damage by ROS it is impossible to predict which patients would be most vulnerable and when. Furthermore, some anaesthetics (e.g., ketamine) have been shown to interfere with mitochondrial function, promote dismutase activity and affect ROS handling, albeit in animal studies (Venâncio et al., 2015).

**FIGURE 7:**
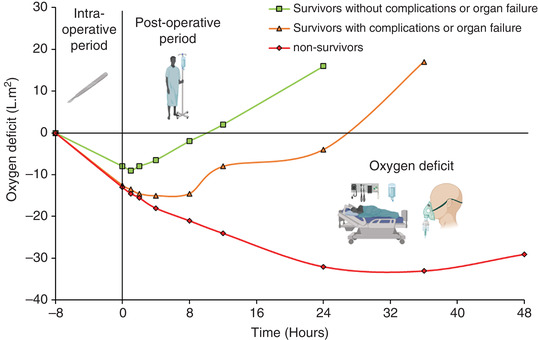
The cumulative O_2_ deficit associated with survivors without complications or organ support, with complications or organ support, and non‐survivors. Adapted from Shoemaker et al. (1992)

**FIGURE 8:**
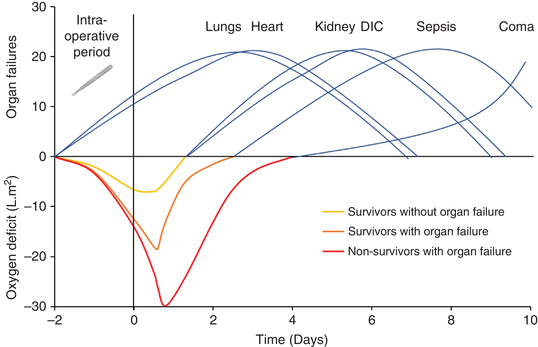
Time course of O_2_ deficit in survivors with and without organ failure and non‐survivors, and the relationship with the emergence and type of organ failures over time, adapted from Shoemaker et al. (1992). Cardiopulmonary complications typically emerge first after surgery, followed by kidney, disseminated intravascular coagulation (DIC), then sepsis and coma

The additional demand for O_2_ is not solely constrained to the intraoperative period. Shoemaker et al. (1992) measured ${\dot{V}}_{O_{2}}$ in 253 high‐risk patients (defined by criteria with a >30% surgical mortality rate) before, during and immediately after major surgery. These values were compared with the estimated ${\dot{V}}_{O_{2}}$ requirements of the patients (using resting preoperative control values) to calculate the magnitude of O_2_ deficit. Patients who died (*n* = 64) had organ failure and a mean O_2_ deficit of 33.2 l m^−2^, compared with 21.6 l m^−2^ for survivors with organ failure (*n* = 31), and 9.2 l m^−2^ for survivors without organ failure or major complications (*n* = 158). These findings highlight the clinical significance of the cumulative O_2_ deficit across the perioperative period and corresponding implications for development of organ failure and ultimately death (Figure 7). Furthermore, the authors also investigated the time course and types of emerging complications up to 10 days following surgery as illustrated in Figure 8. Interestingly, the recovery ‘slopes’ of the O_2_ deficit in Figure 8 are much the same between survivors (with organ failure) and non‐survivors, and just the intraoperative and early postoperative magnitude is greater, which may suggest this to be the more critical component.

## 7 POTENTIAL MECHANISMS THAT ENHANCE SURVIVAL

**FIGURE 9:**
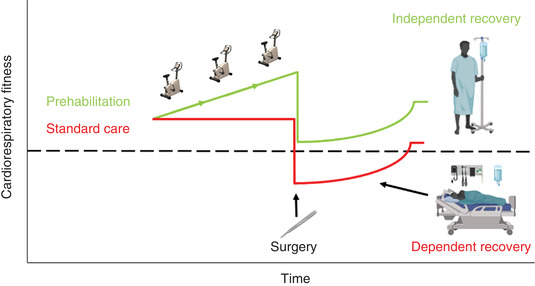
The fundamental principle underlying exercise prehabilitation whereby CRF is improved prior to surgery, thus reducing the risk of postoperative complications, and enhancing recovery as indicted by the green plot. Adapted from Clegg et al. (2013). The dashed line represents the cut‐off between independent (ward‐based care) and dependent (high dependency unit, intensive care unit) patient recovery

Whilst mechanistic bases explaining the link between (elevated) CRF and postoperative outcome require further elucidation, evidence demonstrates that patients with low CRF are associated with poor postoperative outcome, likely explained by the prevailing magnitude of perioperative O_2_ deficit. Importantly, CRF is a modifiable risk factor and a primary component of prehabilitation strategies (Macmillan, 2019; Tew et al., 2018). Prehabilitation represents an opportunity to improve patient preparation for surgery and is multi‐modal in nature comprising exercise training and improving nutritional and psychological status (Scheede‐Bergdahl et al., 2019). Prehabilitation aims to improve patient CRF to better tolerate the surgical stress response, leading to a reduced risk of perioperative complications and improved postoperative outcome (Tew et al., 2018). The theoretical potential for this strategy is illustrated in Figure 9.

Few studies have investigated the potential to improve CRF prior to surgery using exercise interventions and those that have mainly comprise small sample sizes demonstrating proof of principle (Rose et al., 2020; Simonsen et al., 2020). West et al. (2015) deployed an exercise intervention in patients following neoadjuvant chemoradiotherapy prior to surgery. The intervention group comprised 22 patients with 17 controls who followed a high intensity interval training (HIIT) protocol, three times per week for 6 weeks. Following neoadjuvant chemoradiotherapy, ${\dot{V}}_{O_{2}}$ at GET was significantly reduced by a mean of 1.9 ml kg^−1^ min^−1^. Conversely, 6 weeks of subsequent HIIT sessions increased O_2_ uptake at GET by 2.1 ml kg^−1^ min^−1^, whereas it did not change in the controls. In a systematic review, Loughney et al. (2016) concluded that preoperative exercise interventions are safe and feasible, yet there are insufficient controlled trials to draw reliable conclusions about their efficacy and feasibility. Recently, clinical guidelines and recommendations for preoperative exercise training in patients awaiting major non‐cardiac surgery have been published (Tew et al., 2018). However, it is again acknowledged that further research is needed to identify the optimal exercise prescription in different clinical scenarios, particularly in the short preoperative time frame encountered in urgent cancer surgery.

While interest lies in preoperative exercise training, clear translational evidence to improved postoperative outcomes is yet to be established, with studies by West et al. (2015) underpowered for this endpoint. The most current systematic review (of 22 studies) with meta‐analysis claimed that whilst prehabilitation improved preoperative functional capacity (measured by 6‐min walk distance, albeit unlike West et al. (2015) objective measures of CRF including ${\dot{V}}_{O_{2}\mathrm{peak}}$ and GET were not improved) and substantially reduced hospital stay, it did not reduce postoperative complications, 30‐day hospital readmissions or postoperative mortality (Waterland et al., 2021). These findings need to be considered cautiously given the small sample sizes, heterogeneity of exercise interventions, limited reporting of objective measures of CRF, and lack of consensus on standardised endpoints of included studies.

Clearly, there is a requirement for a higher quality of evidence from large, randomised control trials, and clinical trials are ongoing with results awaited. Examples include: Van Rooijen et al. (2019), an international multicentre multimodal prehabilitation intervention including exercise, nutrition and psychological coping strategies within an enhanced recovery after surgery (ERAS) protocol (Trial ID NTR5947); a comparison of hospital‐based supervised exercise, supported home‐based exercise versus usual care to investigate patient recovery after bowel cancer surgery (PREPARE‐ABC, 2020; Trial ID ISRCTN82233115); and Wessex Fit‐4 Cancer Surgery (Southampton University, 2020) investigating the effectiveness of a community‐based structured responsive exercise training programme with or without psychological support (Trial ID NCT03509428).

From a mechanistic perspective, similarities exist between the physiological insult of surgery and the acute response to an exercise stimulus. Primarily, an increased cellular demand for O_2_, consequent to oxidative phosphorylation required to regenerate ATP, is required to enable continued physical activity. As a chronic adaptive response to exercise, an improved ability to increase ${\dot{V}}_{O_{2}}$ is associated with elevated mRNA of peroxisome proliferator‐activated receptor γ coactivator 1‐α (Gibala et al., 2009), a moderator of skeletal muscle mitochondrial biogenesis. An increase in citrate synthase (a marker of muscle oxidative capacity) has also been reported (Burgomaster et al., 2005), and an increase in oxidative stress (Bailey et al., 2010, 2018; Davies et al., 1982; Radák et al., 1999), which is attenuated following exercise training (Fatouros et al., 2004).

The mechanisms of this exercise‐induced response have been linked to improvements in total antioxidant capacity (Fatouros et al., 2004; Radák et al., 1999), which is considered a marker of the body's defence system to neutralise excessive and deleterious free radical and associated ROS formation (Ghiselli et al., 2000). Total antioxidant capacity has been enhanced following exercise training in both animal (Liu et al., 2000) and human (Fatouros et al., 2004) models. However, whether the long‐term exercise‐induced increase in total antioxidant capacity, and thus reduction in oxidative stress, is a key factor in improving postoperative outcomes remains to be elucidated. Exercise training been associated not only with a reduction in oxidative stress, but also with improved vascular function and consequent O_2_ transport (Wray et al., 2011). Systemic and cerebrovascular function has been shown to improve following HIIT (Calverley et al., 2020; Molmen‐Hansen et al., 2012), the potential consequence of an ‘optimised’ blood flow‐shear phenotype, triggering calcium influx into the hyperpolarised endothelial cells (Cooke et al., 1991) upregulating endothelial nitric oxide synthase (Bolduc et al., 2013).

## 8 OPTIMISING RISK QUANTIFICATION AND PATIENT MANAGEMENT

The evidence reviewed suggests that impaired CRF is both an independent and a modifiable risk factor associated with postoperative outcome. Yet the strength of this relationship, used to predict postoperative outcome, is not effectively compared against traditional cardiovascular risk factors such as ischaemic heart disease, lung disease, or diabetes and obesity. This comparison has been addressed epidemiologically for all‐cause deaths (outside of the surgical setting) within the Aerobics Centre Longitudinal Study, in which low CRF was found to be a greater risk factor than hypertension, smoking, high cholesterol, diabetes and obesity (Blair, 2009).

**FIGURE 10:**
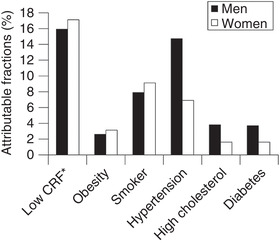
Attributable fractions (%) for all‐cause deaths in the Aerobics Centre Longitudinal Study, taken from Blair (2009). *Cardiorespiratory fitness determined by a maximal exercise test on a treadmill

Attributable fractions describe the percentage of deaths that would not occur if a risk factor were removed from a population and account for both the risk of mortality associated with that condition and its prevalence in the population, as illustrated in Figure 10. This approach could be conducted in the surgical setting to help optimise risk quantification and further highlight the clinical importance of CRF relative to traditional risk factors.

Like all biomarkers, CRF is a dynamic metric subject to natural variation and thus needs to be interpreted with caution. Such variation encompasses both analytical and biological components which can be described using the concept of critical difference, indicative of the magnitude of variation around a true homeostatic point at any given time. Rose et al. (2018b) introduced the concept of critical difference to preoperative CPET and found differences of ± 19%, 13% and 10% for ${\dot{V}}_{O_{2}}$‐GET, ${\dot{V}}_{O_{2}\mathrm{peak}}$ and $V_{\mathrm{eqC}O_{2}}$‐GET. The translational impact upon patient fitness stratification in their study demonstrated that up to 60% of patients were of indeterminate fitness, where for example, they could not be sure that a patient had a ‘true’ GET < 11 ml O_2_ kg^−1^ min^−1^ when variation was accounted for. A revised stratification model was formulated using zones along a spectrum of fitness; thus, clinicians are advised to look beyond a single cut‐point and instead advocate a dynamic range of CPET values indicative of surgical risk (Wilson, 2018).

Furthermore, whilst inter‐observer agreement, using intra‐class correlation coefficient (ICC), for numerical values of GET (ICC 0.83 (0.75–0.90)) and ${\dot{V}}_{O_{2}\mathrm{peak}}$ (ICC 0.88 (0.84–0.92)) indicating good to excellent relative reliability (Abbott et al., 2018), inter‐observer agreement regarding whether or not a reportable value existed was less consistent. This suggests that guidance for identification of reportable values could be improved.

Patient stratification should be optimised using the most effective metrics of CRF, with accompanying threshold values, which are indicative of risk specific to patient populations and surgical procedures. Table 1 highlights that many studies, including the seminal work of Older et al. (1993), have simply adopted threshold values developed by other studies sometimes using different patient populations and surgical procedures. Furthermore, CRF is commonly described using ${\dot{V}}_{O_{2}\mathrm{peak}}$, GET or $V_{\mathrm{eqC}O_{2}}$ as discussed; however, alternative metrics may provide superior prognostic utility in some settings. For example, if a patient is unable or unwilling to exercise to exhaustion, a submaximal measure of CRF relating O_2_ consumption to workload achieved, such as the O_2_ uptake efficiency slope (OUES; Hollenberg & Tager, 2000; Bongers et al., 2017), may be more effective.

Female inclusion rate in peer‐reviewed publications of perioperative CPET is reported at only 31% and may have a bearing on the interpretation of data (Thomas et al., 2020). Surprisingly, despite evidence that CRF is lower in females across the lifespan, given smaller body size, skeletal muscle mass, peak cardiac output and Hb concentration (Jackson et al., 2009; Fleg et al., 2005), sex is not considered during surgical risk stratification. If a simple dose–response relationship exists between low CRF and postoperative survival, we would expect females to be at increased risk given these congenital constraints. Furthermore, other risk factors such as cardiovascular disease (CVD), which may vary between the sexes, require investigation to appraise a potential compensatory effect for CRF and consequent changes in its prognostic potential on postoperative outcome.

## 9 CONCLUSION

The current review has explored the intimate relationship between O_2_ transport and postoperative outcome, emphasising how preoperative CRF is an independent risk factor for postoperative mortality and morbidity, when patients undergo major intra‐abdominal surgery. There is increased O_2_ demand during the perioperative period and patients must meet this demand to avoid tissue hypoxia, the presence and magnitude of which dictates postoperative morbidity and mortality. This relationship can be used to assess patient risk, plan perioperative care and optimise patient management using exercise as a modifiable intervention. However, there is a clear need to improve the physiological detection and interpretation of CRF, better quantify risk to specific populations, sex and surgical procedure, and better understand the optimal management of patients including the mode of exercise intervention and its timing. Collectively, a better understanding of CRF used to determine fitness for surgery will enable clinicians and physiologists alike to direct patient care more effectively and ultimately improve survival.

## COMPETING INTERESTS

D.M.B. is Chair of the Life Sciences Working Group and member of the Human Spaceflight and Exploration Science Advisory Committee to the European Space Agency and member of the Space Exploration Advisory Committee to the UK Space Agency.

## AUTHOR CONTRIBUTIONS

All authors have read and approved the final version of this manuscript and agree to be accountable for all aspects of the work in ensuring that questions related to the accuracy or integrity of any part of the work are appropriately investigated and resolved. All persons designated as authors qualify for authorship, and all those who qualify for authorship are listed.
